# Memory consolidation in humans: new evidence and opportunities

**DOI:** 10.1113/expphysiol.2013.072157

**Published:** 2014-03-04

**Authors:** Eleanor A Maguire

**Affiliations:** Wellcome Trust Centre for Neuroimaging, Institute of Neurology, University College LondonUK

## Abstract

We are endlessly fascinated by memory; we desire to improve it and fear its loss. While it has long been recognized that brain regions such as the hippocampus are vital for supporting memories of our past experiences (autobiographical memories), we still lack fundamental knowledge about the mechanisms involved. This is because the study of specific neural signatures of autobiographical memories *in vivo* in humans presents a significant challenge. However, recent developments in high-resolution structural and functional magnetic resonance imaging coupled with advanced analytical methods now permit access to the neural substrates of memory representations that has hitherto been precluded in humans. Here, I describe how the application of ‘decoding’ techniques to brain-imaging data is beginning to disclose how individual autobiographical memory representations evolve over time, deepening our understanding of systems-level consolidation. In particular, this prompts new questions about the roles of the hippocampus and ventromedial prefrontal cortex and offers new opportunities to interrogate the elusive memory trace that has for so long confounded neuroscientists.

## Introduction

**Figure 1:**
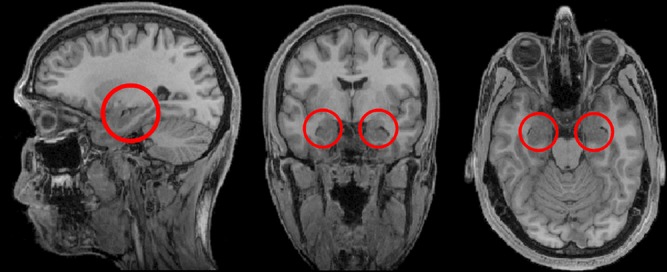
The hippocampi are circled in red on sagittal (left), coronal (middle) and axial views (right) from a T1-weighted structural magnetic resonance imaging (MRI) brain scan.

There are many brain regions that co-operate to enable us to form, store and retrieve memories of our personal past experiences, which are known as autobiographical memories (Maguire, 2001; Svoboda *et al*. 2006). However, there is one undoubted star of the show and that is the hippocampus, buried deep in the brain's temporal lobes (Fig. 1). The consequences of hippocampal damage can be devastating and far-reaching, not only robbing us of our past (Scoville & Milner, 1957), but preventing us from inhabiting our imagination (Hassabis *et al*. 2007*b*; Mullally *et al*. 2012) and contemplating the future (Tulving, 1985; Klein *et al*. 2002; Hassabis *et al*. 2007*b*; Rosenbaum *et al*. 2009; Andelman *et al*. 2010; Race *et al*. 2011), as well as impairing the ability to navigate effectively in the environment (Maguire *et al*. 2006) and even altering our perception of the world (Graham *et al*. 2010; Lee *et al*. 2012; Mullally *et al*. 2012). The hippocampus therefore seems to make fundamental contributions to our everyday mental experience, but there is still much we do not know about how it accomplishes these feats.

Models of memory posit the existence of some form of memory representation within the hippocampus that is vital for retrieving an entire memory, the constituent elements of which may be distributed in the cortex (Marr, 1971; O'Reilly & McClelland, 1994; Treves & Rolls, 1994; McClelland & Goddard, 1996; O'Reilly & Rudy, 2000). The theoretical nature of this hippocampal memory trace has been modelled over the years, and related computations have been extensively studied in the rodent hippocampus (Vazdarjanova & Guzowski, 2004; Lee *et al*. 2004; Leutgeb *et al*. 2004, 2007; Wills *et al*. 2005). While unquestionably offering insights into memory representations, it is unknown whether non-humans possess the capacity for autobiographical memory (Suddendorf, 2013; but see Clayton *et al*. 2003; Corballis, 2013), rendering the relevance of this work for complex autobiographical memories in humans unclear.

In contrast, humans are undeniably adept at recalling and describing their past experiences, but studies have lacked the precision of animal work in trying to elucidate how individual autobiographical memories are represented by neuronal populations in the human hippocampus. This is, in part, a result of the dominant methodological approaches in human cognitive neuroscience. Neuropsychological studies of patients with bilateral hippocampal lesions have been invaluable for mapping out the specific patterns of mnemonic sparing and deficits that arise following such damage (Scoville & Milner, 1957; Mayes, 1988; Spiers *et al*. 2001; Winocur & Moscovitch, 2011). Conventional functional neuroimaging, such as functional magnetic resonance imaging (fMRI), has also proved effective for localizing a wide range of cognitive functions to specific brain regions while also highlighting the importance of network activity (e.g. Bullmore & Sporns, 2009; Yarkoni *et al*. 2011). However, neither neuropsychology nor conventional fMRI can inform directly about the neuronal representation of specific autobiographical memory traces.

This is problematic, because some of the key questions about memory are best leveraged by examining individual memory representations. However, in recent years new ways of analysing fMRI data have emerged. Here, using one of these methods (for others see Chadwick *et al*. 2012), I will describe how it is now possible to detect specific memory representations *in vivo* in the human brain, and how we can use this ability to gain new insights into an issue at the heart of memory neuroscience, namely, how do autobiographical memories evolve over time, a process known as systems-level consolidation (Dudai, 2004, 2012; Nadel *et al*. 2007*b*2007b, 2012).

## Multi-voxel pattern analysis

**Figure 2:**
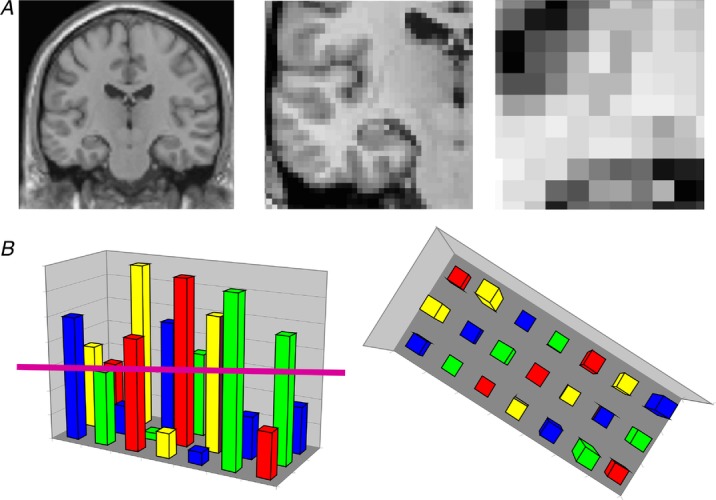
*A*, the left panel shows a coronal section through an MRI brain scan. Zooming in progressively further (middle and then right panels), the voxels that make up the scan are evident. *B*, the left panel shows a schematic diagram of some voxels from a functional magnetic resonance imaging (fMRI) brain scan. The pink line indicates the statistical threshold for activation, with some of the voxels passing the threshold and others not. With this conventional approach to analysing fMRI data, each voxel is considered separately. The right panel shows another view (from overhead) of the same set of voxels. A pattern across voxels is now evident. This focus on patterns of fMRI activity across voxels is known as multi-voxel pattern analysis (MVPA).

In a conventional fMRI analysis, the responses to memories of the same type (for example, a set of autobiographical memories) are pooled together. We cannot normally examine individual memories because the fMRI signal would be insufficient; hence, we average across a group of memories. The response to that memory type is assessed in each voxel – a voxel being the smallest measureable unit in a three-dimensional brain image volume (Fig. 2*A*) – to establish which individual voxels pass an *a priori* statistical threshold of activation (Fig. 2*B*). In this approach, therefore, every voxel is considered separately. However, important findings could be missed from conventional fMRI analyses if information is represented in distinct patterns across voxels and, by inference, across the underlying neuronal population, rather than in the number of separate individual voxels that reach a threshold of activation (Fig. 2*B*). Analysing fMRI data in terms of the activity patterns across multiple voxels is known as multi-voxel pattern analysis (MVPA; Haynes & Rees, 2006; Norman *et al*. 2006).

In an MVPA analysis, an fMRI data set is split into two; one part is set aside, and the other is used to train a computer algorithm, or classifier, to learn over multiple trials the patterns of activity across voxels that are associated with specific stimuli. The classifier is then applied to the data that were not used for training to ascertain whether it can predict significantly better than chance which specific stimulus the participant had been processing during that part of the fMRI experiment. If the classifier is successful at predicting which stimulus the participant was processing, it must mean that there is information about that stimulus represented in the brain region where the pattern of voxels was identified. Crucially, this type of approach permits examination of patterns of fMRI activity associated with specific stimuli.

With that in mind, using MVPA we found that it was possible to predict a participant's precise spatial location within a virtual reality environment solely from the patterns of fMRI activity across voxels in the hippocampus (Hassabis *et al*. 2009). In another study, participants watched short movie clips prior to entering the scanner, and then during scanning had to recall the movie clips. Once again, it was possible to predict which specific movie a participant was recalling solely from the pattern of fMRI activity across voxels in the hippocampus (Chadwick *et al*. 2010). In these studies, the items to be remembered were relatively simple and were provided by the experimenters. Autobiographical memories, in contrast, are rich and complex and are unique to each person. As such, they might be expected to pose a substantial challenge for MVPA classifiers. Would it really be possible to predict which specific autobiographical memory a person is recalling solely from patterns of fMRI activity?

## Theoretical considerations

This question begs another. Are events that we recently experienced represented in a similar or different way by the hippocampus compared with autobiographical experiences from long ago? Consolidation of memories undoubtedly occurs rapidly at the synaptic level (Dudai, 2004). In contrast, systems-level consolidation (Dudai, 2004, 2012) and how the neural instantiation of autobiographical memories might change over longer time scales remain uncertain. In general, extant theories agree that the cortex comes to play a greater role in supporting autobiographical memories over time (Marr, 1971; Teyler & DiScenna, 1985; Squire, 1992; Nadel & Moscovitch, 1997; Squire & Wixted, 2011; Winocur & Moscovitch, 2011; Nadel *et al*. 2012). The precise areas of cortex that may be involved are often not specified, although the ventromedial prefrontal cortex (vmPFC), in particular, has been highlighted as potentially influential for memory consolidation (Bontempi *et al*. 1999; Frankland & Bontempi, 2005; reviewed by Nieuwenhuis & Takashima, 2011).

**Figure 3:**
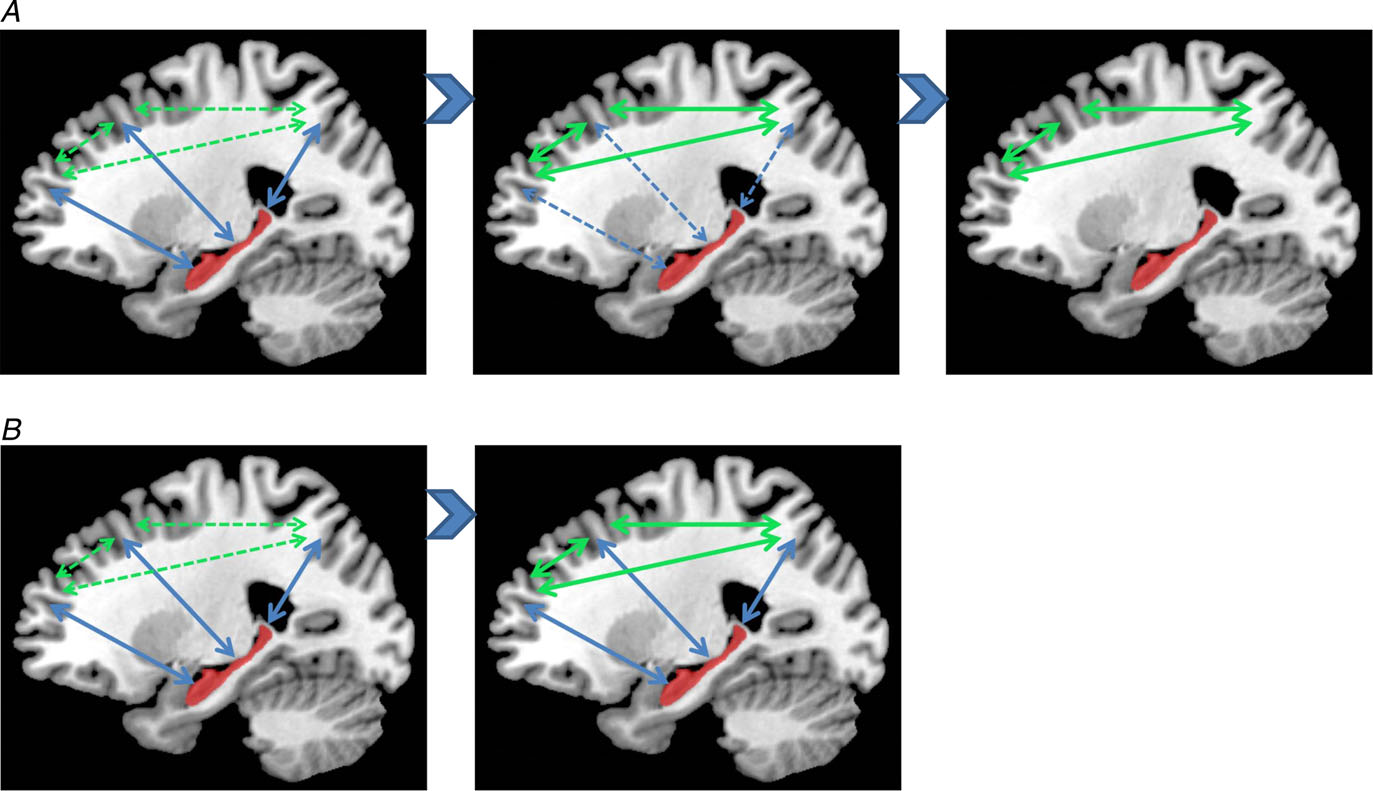
*A*, a schematic diagram of the standard model of consolidation shown on sagittal brain slices, where information is first processed by the hippocampus and progressively, over time, the cortico-cortical connections become stronger so that, finally, the memory is represented in the cortex and the hippocampus is not required at retrieval. *B*, alternative theories also posit that information is first processed by the hippocampus and progressively, over time, the cortico-cortical connections become stronger. However, the input of the hippocampus is still required for vividly retrieving autobiographical memories, even those that were formed many years ago.

There is also agreement that autobiographical memories depend on the hippocampus during initial encoding (Scoville & Milner, 1957). However, its role in supporting autobiographical memories when they are more remote is contentious. The standard model of consolidation (Fig. 3*A*) argues that memories (including autobiographical memories) become less dependent on the hippocampus, eventually eschewing the need for its involvement altogether during retrieval (Marr, 1971; Teyler & DiScenna, 1985; Squire, 1992; Squire & Wixted, 2011). Alternative theories (multiple trace theory, scene construction theory; Fig. 3*B*) propose instead that the hippocampus is necessary for retrieving vivid autobiographical memories in perpetuity (Nadel & Moscovitch, 1997; Hassabis & Maguire, 2007, 2009; Winocur & Moscovitch, 2011; Maguire & Mullally, 2013). There is neuropsychological evidence from patients with bilateral hippocampal damage, including those with lesions apparently restricted to the hippocampi, in support of both views (for a review see Winocur & Moscovitch, 2011). Some patients have a limited retrograde amnesia (that is, amnesia for experiences that occurred prior to lesion onset), supporting the standard model of consolidation, because it predicts an impairment of recent memories that might still be dependent on the hippocampus, but sparing of remote memories, which are available in the undamaged cortex. In contrast, other patients are reported who are amnesic for events throughout their lifetime, suggesting that the hippocampus is vital for retrieving even very remote autobiographical memories. Conventional fMRI studies exploring this issue have also produced conflicting findings (e.g. Maguire *et al*. 2001; Ryan *et al*. 2001; Maguire & Frith, 2003; Gilboa *et al*. 2004; Piolino *et al*. 2004; Rekkas & Constable, 2005; Steinvorth *et al*. 2006; Viard *et al*. 2007; Watanabe *et al*. 2012; but see Niki & Luo, 2002; Piefke *et al*. 2003) and so contribute to the stalemate.

The key question in this debate, when reduced to its simplest form, concerns where information about specific autobiographical memories is located. Multi-voxel pattern analysis seems well suited to addressing this question and adjudicating between the opposing theories. The standard consolidation view predicts that recent autobiographical memories are represented in the hippocampus and remote autobiographical memories in the cortex. Alternative theories predict that for vivid autobiographical memories, both recent and remote memories are represented in the hippocampus, while the cortex contains more information about remote autobiographical memories.

## Detecting autobiographical memory representations

In a recent study, we tested these predictions using MVPA (Bonnici *et al*. 2012). A week prior to scanning, each subject was interviewed to ascertain details of numerous autobiographical memories. From this pool of memories, three recent memories that were 2 weeks old and three remote memories that were 10 years old were selected for inclusion in the fMRI experiment. From the descriptions of these memories provided by participants we could discern no differences between the recent and remote autobiographical memories. Specifically, great care was taken to ensure that the recent and remote memories were re-experienced with equal and high vividness. In addition, based on analysis of the memory descriptions and ratings provided by the participants, the frequency of recall, level of detail, emotional valence and the perspective taken were all matched between recent and remote memories. We also made strenuous efforts to study memories that were unique, excluding events that occurred repeatedly or were similar to other events. We confirmed that subjects did not think about the previous week's stimulus-eliciting interview at all during scanning. From an intensely interrogated phenomenological perspective, therefore, the recent and remote memories did not differ in any measureable way. During scanning, subjects recalled the memories, and after each recall trial rated whether or not the memory was recalled with high vividness, with only vividly recalled memories included in the MVPA analysis.

**Figure 4:**
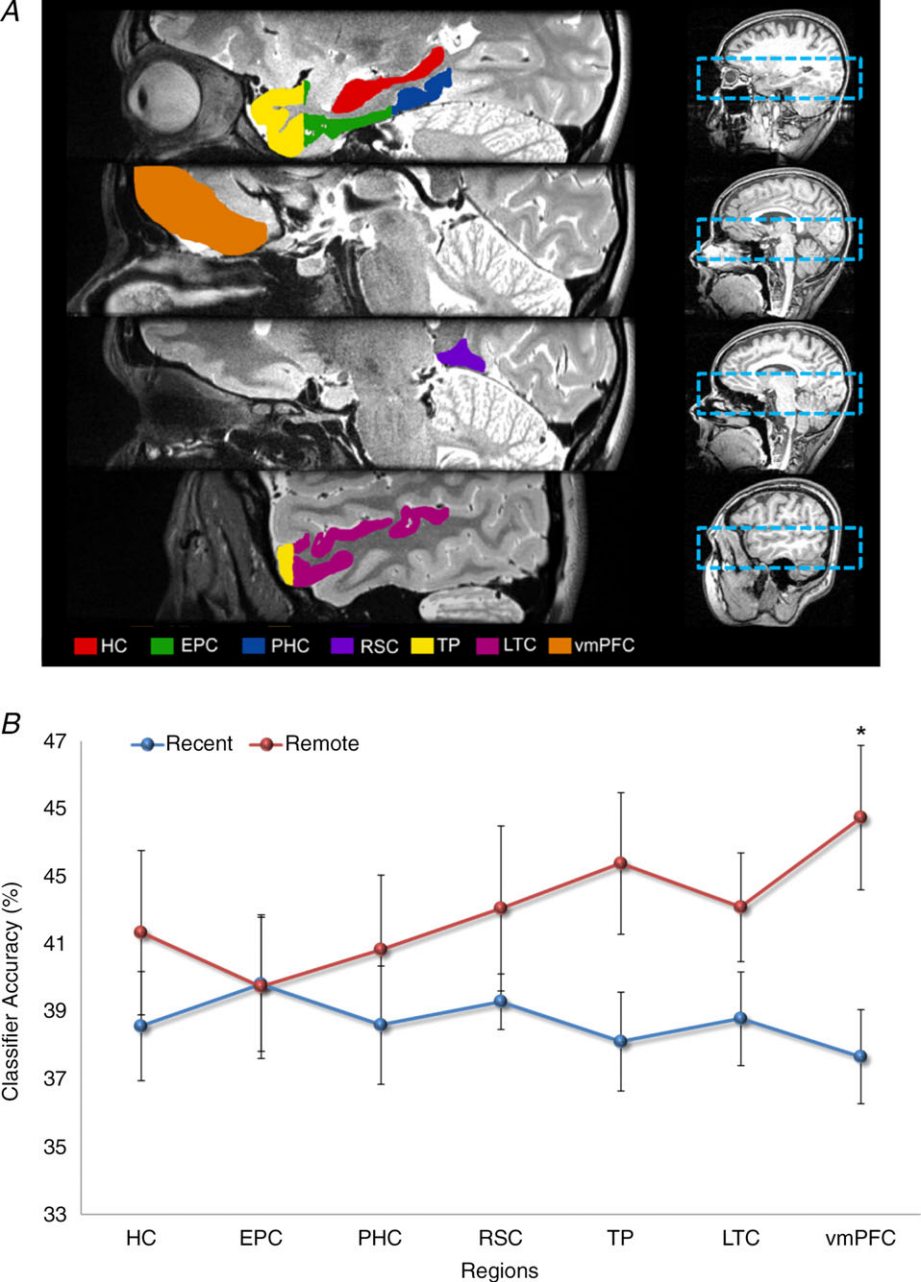
*A*, the brain areas examined by Bonnici *et al*. (2012). The right panels show the bounding box of the high-resolution partial volume that was acquired for every subject. The left panels show the regions of interest that were demarcated, namely: hippocampus (HC), entorhinal and perirhinal cortices (EPC; combined because their responses were so similar), parahippocampal cortex (PHC), retrosplenial cortex (RSC), temporal pole (TP), lateral temporal cortex (LTC) and ventromedial prefrontal cortex (vmPFC). *B*, the MVPA results for memory decoding in each of the demarcated brain regions for recently formed autobiographical memories (blue) and for autobiographical memories that were formed 10 years ago (red). There was no significant difference in the classifier accuracy values for recent and remote memories in the hippocampus, but in vmPFC there was more accurate decoding of remote memories compared with recent memories (data from Bonnici *et al*. 2012; *P < 0.05; chance is 33%).

As well as the hippocampus, a number of cortical brain areas were examined, namely, entorhinal and perirhinal cortices, parahippocampal and retrosplenial cortices, temporal pole, lateral temporal cortex and vmPFC (Fig. 4*A*). As shown in Fig. 4*B*, it was possible to predict which recent and also which remote autobiographical memory was being recalled significantly above chance in all brain areas, underlining the distributed nature of complex autobiographical memory representations (Lashley, 1950). Notably, there was no significant difference in the classifiers’ performances for decoding recent and remote memories in the hippocampus. In contrast, vmPFC contained more information about remote autobiographical memories compared with recent memories.

**Figure 5:**
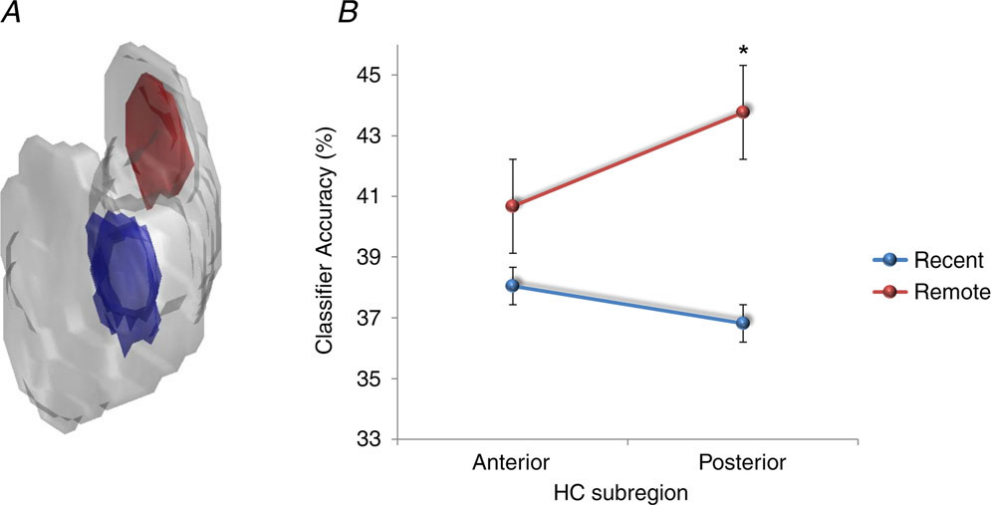
*A*, the voxels that best differentiated between the recent autobiographical memories (blue) and the voxels that best differentiated between the remote autobiographical memories (red) were reprojected back into the hippocampus. One example three-dimensionally rendered hippocampus from one subject illustrates clearly the lack of overlap between them. *B*, when the hippocampus was split into its anterior and posterior portions, there was no significant difference between classifier accuracies for recent and remote memories in the anterior part. However, remote memories were better represented in the posterior hippocampus compared with recent memories (data from Bonnici *et al*. 2012; *P < 0.05; chance is 33%).

There were two other interesting findings. Although the hippocampus seemed to represent recent and remote memories in a similar manner, when the voxels that best discriminated between the recent memories and those that best discriminated between the remote memories were reprojected back into the hippocampi, it quickly became clear that the clusters of voxels hardly overlapped (Fig. 5*A*). This was confirmed by a formal analysis, which showed that the overlap for recent and remote memory reprojections was significantly below chance for the hippocampus. This was in contrast to cortical areas, such as vmPFC, where the voxel patterns (and by inference the underlying neuronal populations) that supported the recent memories overlapped with those supporting the remote memories. Moreover, the location of recent and remote voxel patterns had a systematic distribution down the long axis of the hippocampus. In the anterior hippocampus, there was no significant difference in classification accuracies for the two types of memories. In contrast, classification accuracies were significantly higher in the posterior hippocampus for remote memories compared with recent memories (Fig. 5*B*). Thus, exactly as with vmPFC, the hippocampus too seems to respect the distinction between recent and remote autobiographical memories.

In interpreting these results, it is important to consider whether any other factors besides the recency/remoteness of autobiographical memories could have influenced the results. For instance, during scanning perhaps participants were recalling the prescan interview where the memories were initially elicited. However, when asked how often they had thought about the memories since the session a week earlier, their ratings confirmed that they had hardly given them any thought. Moreover, the interview concerned both the recent and remote memories, and so the differential effects for the two types of memories that we found could not have arisen from this common interview experience.

The scanning protocol required participants to recall the memories a number of times during scanning, and it could be argued that the memories were re-encoded on each trial, which may have polluted the recall effects. As with the interview above, if re-encoding did occur, it would presumably have done so for both recent and remote memories, again making the differential results we found difficult to explain. In addition, the nature of MVPA means that classification is only possible if information is shared across training and test trials. Re-encoded memories would have been different for each trial, leading to chance classification, which was not the case here. On a related theme, it could have been that recalling a remote memory reactivated it, effectively transforming it back into a recent memory. If this were the case, then the prediction would be for no difference between recent and remote memories (if all memories were now essentially recent). However, the differential effects, cortically and within the hippocampus itself, clearly contradict this idea.

Repeatedly recalling the memories may have had other effects. For instance, it may have lessened the true episodic nature of the memories and influenced hippocampal engagement. However, previous studies have shown that hippocampal activation does not diminish as a function of multiple retrievals of autobiographical memories (Nadel *et al*. 2007*a*2007a; Svoboda & Levine, 2009). The participants also confirmed that repeated recall did not change the memory, and only those trials where participants indicated that they had recalled the memory with high vividness were included in the analysis. Given that ease of recall and other phenomenological factors were also highly similar across the recent and remote memories, neither these, nor the alternatives above, adequately explain the differential findings.

Recently, Bonnici and Maguire (in preparation) rescanned the subjects from Bonnici *et al*. (2012) to examine how the memories in question had evolved in the intervening 2 years (H.M. Bonnici & E.A. Maguire, unpublished observations). They found that the now 2- and 12-year-old memories were indistinguishable in terms of involvement of the vmPFC, while within the hippocampus, the 2-year-old memories were represented more posteriorly than they were 2 years previously. This is despite the vividness and other phenomenological aspects of the memories remaining essentially unchanged.

**Figure 6:**
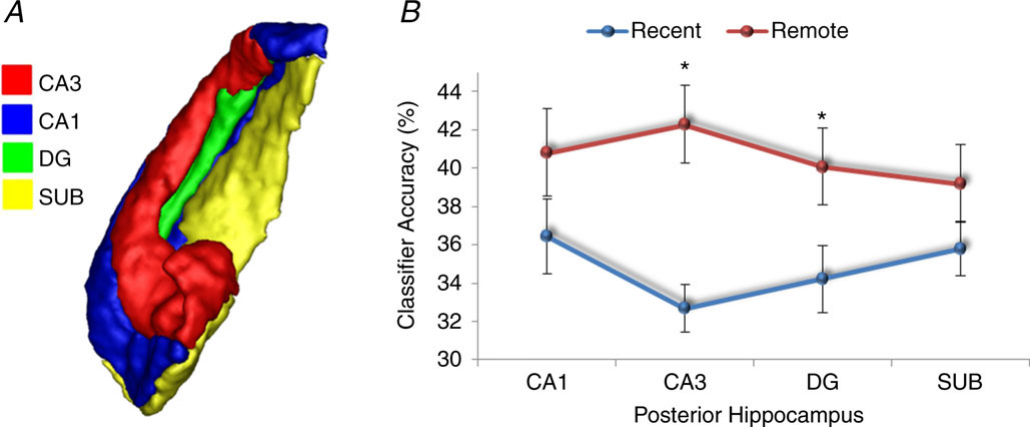
*A*, an example hippocampus shown divided into its constituent subfields, CA1, CA3, dentate gyrus (DG) and subiculum (SUB). *B*, in the anterior hippocampus, there was no significant difference between classifier accuracies for recent and remote autobiographical memories in any subfield. In contrast, in the posterior hippocampus there was more information represented in CA3 and DG for remote compared with recent memories (data from Bonnici *et al*. 2013*a*2013a; *P < 0.05; chance is 33%).

As well as having anterior and posterior portions, the hippocampus is composed of a number of subregions, i.e. CA1, CA2 and CA3 (Lorente de No, 1934), bordered by the dentate gyrus (DG) and subiculum (Amaral & Lavenex, 2007; Fig. 6*A*). The findings of Bonnici *et al*. (2012) gave no indication of whether the anterior–posterior differential effects were being driven by all subfields or by one or two in particular. In a recent study, therefore, Bonnici *et al*. (2013*a*), using high-resolution fMRI combined with MVPA, found that it was possible to detect representations of specific autobiographical memories in individual hippocampal subfields. Moreover, while subfields in the anterior hippocampus contained information about both recent and remote autobiographical memories, posterior CA3 and DG contained only information about the remote memories (Fig. 6*B*). Thus, the subfields in posterior hippocampus seem to be involved differentially in the representation of recent and remote autobiographical memories during vivid recall.

## Re-examining the role of the hippocampus

This work shows that despite being rich, detailed and complex, using MVPA it is possible to predict which autobiographical memory a person is recalling solely from patterns of fMRI activity across voxels. This implies that information about recent and remote autobiographical memories is represented in the hippocampus, while within the cortex remote memories are better represented, in particular in vmPFC. These findings clearly support the alternative theories of consolidation (Nadel & Moscovitch, 1997; Hassabis & Maguire, 2007, 2009; Winocur & Moscovitch, 2011; Maguire & Mullally, 2013; Fig. 3*B*), but the findings of Bonnici *et al*. (2012, 2013*a*) go further and raise some interesting new issues about the functioning of the hippocampus.

While the overall performance of the hippocampal classifiers for recent and remote memories was similar, when the voxel patterns were examined an anterior–posterior distinction emerged, with the posterior hippocampus representing remote autobiographical memories more than recent. This suggests that as well as consolidation occurring within the cortex (vmPFC), there are changes within the hippocampus also. Moreover, this may account for the disparate findings across patients with hippocampal damage; if a lesion encroaches more on posterior hippocampus, then remote autobiographical memories may be more compromised than if damage was more anterior (Penfield & Mathieson, 1974; Winocur & Moscovitch, 2011). While Gilboa *et al*. (2004) suggested that remote autobiographical memories might be distributed throughout the hippocampus, Bonnici *et al*. (2012) have shown that there is a clear anterior–posterior distinction that is not predicted by any extant theory. Indeed, when Bonnici *et al*. (2012) pooled across all memories they found it was possible to classify any given memory as recent or remote solely from fMRI activity across voxels in the hippocampus. This suggests that something is signalling that a memory is recent or remote; after all, people can readily distinguish between recent and remote autobiographical memories, suggesting that there is something different about them, but what might this be?

As described earlier, the recent and remote memories were scrupulously matched on a wide range of variables; they were all vividly recalled, and it was not that the remote memories were more semanticised, at least at an explicit, phenomenological level. When the memories were retested 2 years later, the representations of the memories that had been a few weeks old but were now some years older differed in terms of the locations of discriminating voxels, with a shift more posteriorly in the hippocampus. This is despite being the same memories, with the same content and similar ratings for vividness and other key variables. One explanation for this finding could be that overlap is still present in some of the initial neuronal connections, but that synaptic pruning of weakly connected neurons resulted in an overall decrease in activity in the original retrieval network, and a reorganization of the memory network involved expansion to include a broader range of connections over time. Alternatively, the shift of autobiographical memory representations from one end of the hippocampus to another, regions that are anatomically distinct and with dissimilar connectivity, could indicate that the recall of autobiographical memories does not necessarily involve the re-instantiation of ensemble activity in the same neurons that were involved when the memories were young. Thus, the traditional view of ‘cells that fire together, wire together’ (Hebb, 1949) may not apply in this case. It is impossible to adjudicate between these explanations currently, but further developments in high-resolution fMRI and multivariate analysis methods may make this issue tractable in the future.

The discovery that the anterior and posterior hippocampus might have different functions has been of increasing interest in the last few years. Poppenk *et al*. (2013) recently reviewed numerous reports of differential engagement of anterior and posterior hippocampus in humans and other species. They suggested that the posterior hippocampus may represent more fine-grained, detailed memories, whereas the anterior hippocampus might support more gist-like retrieval (see also Evensmoen *et al*. 2013). However, given that both recent and remote autobiographical memories were rich and detailed and, if anything, recent memories might be expected to be richer and more detailed (although Bonnici *et al*. 2012 ensured that this was not the case at an explicit level), then it is difficult to explain the propensity of the posterior hippocampus to represent specifically remote autobiographical memories.

The posterior hippocampus has been associated with spatial processing (e.g. Moser & Moser, 1998; Maguire *et al*. 2000). Perhaps recent memories are experienced as coherent scenes or events that are temporarily represented in the hippocampus (using anterior and posterior aspects), with cortical consolidation happening relatively quickly. The constituent elements of autobiographical memories are then the preserve of the cortical areas. At retrieval, this piecemeal information might be funnelled back automatically into the hippocampus (in a process that may involve vmPFC), but in order to be assembled into a coherent form, this requires a process that takes place in the posterior hippocampus (Bonnici *et al*. 2012). This may be why the remote memories were discernible to a greater degree in posterior hippocampus, because they rely on this process more than do recent memories. A further speculation might be that the posterior hippocampus may implement the spatial framework into which the elements of a remote memory are reconstructed (Hassabis & Maguire, 2007, 2009; Maguire & Mullally, 2013), in line with findings from patients with hippocampal damage who have lost the ability to construct spatially coherent scenes (e.g. Hassabis *et al*. 2007b; Race *et al*. 2011; Mullally *et al*. 2012).

Studies in rodents and computational models suggest that key computations necessary for memory occur in the subfields, such as pattern separation (in DG and CA3), the process of distinguishing similar memories from each other, as well as pattern completion (in CA3), which facilitates the retrieval of previously stored memories from partial cues (Marr, 1971; Treves & Rolls, 1994; McClelland *et al*. 1995; Kesner *et al*. 2004; Leutgeb *et al*. 2004, 2007; Leutgeb & Leutgeb, 2007; Alvernhe *et al*. 2008; Hunsaker & Kesner, 2008; Gilbert & Brushfield, 2009; Rolls, 2010; Aimone *et al*. 2011; O'Reilly *et al*. 2011). Bonnici *et al*. (2013*a*2013a) noted the particular involvement of CA3 and DG subfields in the posterior hippocampus during recall of remote autobiographical memories. If remote memories have to undergo more reconstruction than recent memories, then the accumulation of memory elements and spatial contexts in the posterior hippocampus might trigger CA3-mediated pattern completion to a greater extent, while DG might act to ensure that this reconstructed memory is maintained as distinct from other memories that the hippocampus might be processing at that time or related memories that might be partly coactivated during retrieval.

## Gaps in our knowledge about vmPFC function

**Figure 7:**
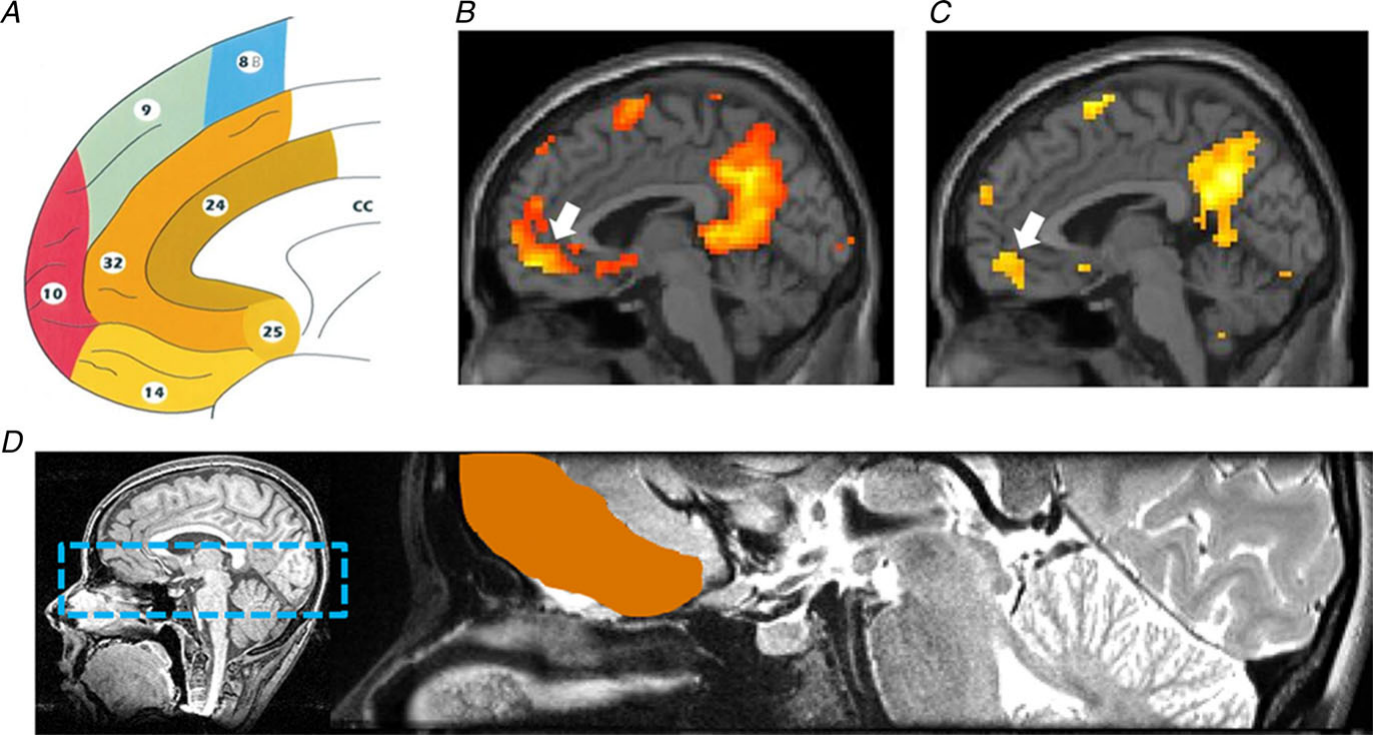
*A*, a map of the human medial frontal cortex from Petrides & Pandya (1999; reproduced with permission from John Wiley & Sons). Examples of two conventional fMRI studies showing activity associated with autobiographical memory retrieval from Hassabis *et al*. (2007*a*2007a) in *B* and from Summerfield *et al*. (2009) in *C*. White arrows indicate the location of the activation in vmPFC that includes part of area 14, ventral parts of 24 and 32, the caudal part of area 10, and also some involvement of area 25. *D*, the vmPFC region analysed in the MVPA study of Bonnici *et al*. (2012) included these areas and more, specifically all of areas 14 and 25, ventral parts of areas 10, 24 and 32, and medial parts of area 11. The left panel shows the bounding box within which data were acquired on a T1-weighted structural MRI brain scan, and the right panel shows a close-up of an example T2-weighted structural MRI brain scan with the vmPFC region of interest delineated in orange.

All of the cortical regions tested by Bonnici *et al*. (2012) showed above-chance decoding of both recent and remote autobiographical memories. However, the vmPFC was alone in displaying significantly better decoding for remote autobiographical memories. The vmPFC is consistently engaged during autobiographical memory retrieval in conventional fMRI studies. However, there is some variation in the cortical areas labelled as vmPFC in the literature. Using the scheme of Petrides & Pandya (1999; Fig. 7*A*), I am referring to portions of area 14, ventral parts of 24 and 32, the caudal part of area 10, the medial part of 11, with additional involvement of area 25 (see examples in Fig. 7*B* and *C*). Of note, these frontal areas are known to be heavily interconnected with each other (Saleem *et al*. 2008; Beckmann *et al*. 2009; Passingham & Wise, 2012); hence, Bonnici *et al*. (2012) examined them altogether in their vmPFC region of interest (Fig. 7*D*). This vmPFC region has also been highlighted as potentially influential for memory consolidation in other studies (e.g. Bontempi *et al*. 1999; Frankland & Bontempi, 2005; Goshen *et al*. 2011; Nieuwenhuis & Takashima, 2011).

The MVPA data revealed a particular association between remote autobiographical memories and the vmPFC. Moreover, Bonnici *et al*. (2012) found that recent and remote autobiographical memories were not represented in different parts of the vmPFC region of interest. In contrast to the hippocampus, the discriminating voxels for recent and remote memories overlapped within vmPFC, suggesting that they may share underlying neuronal ensembles. Nieuwenhuis & Takashima (2011) proposed that the vmPFC may link cortical representational areas in remote memory because, over time, direct connectivity with limbic regions, such as the hippocampus, decays (Fig. 3*A*). However, the idea that the vmPFC assumes the role of the hippocampus over time is not supported by the MVPA findings, where remote memories were still represented in the hippocampus as well as the vmPFC. An alternative view, which accords with the proposal of Winocur & Moscovitch (2011), is that perhaps the quality of the remote autobiographical memory representations in vmPFC differ from those in the hippocampus. It could be that the initial retrieval of a remote memory reactivates both a schematic, gist-like, cortical version and a detailed hippocampal version of the memory. As already noted, at an explicit and phenomenological level the recent and remote autobiographical memories in the Bonnici *et al*. (2012) study were of equivalent vividness and detail, which speaks against this view. Moreover, whether there is a need for two versions of the same memory, particularly when one is detailed, seems somewhat unlikely, because this redundancy would be an inefficient use of neural resources. Perhaps examination of the wider literature on vmPFC might offer some guidance on interpreting the MVPA autobiographical memory findings.

**Figure 8:**
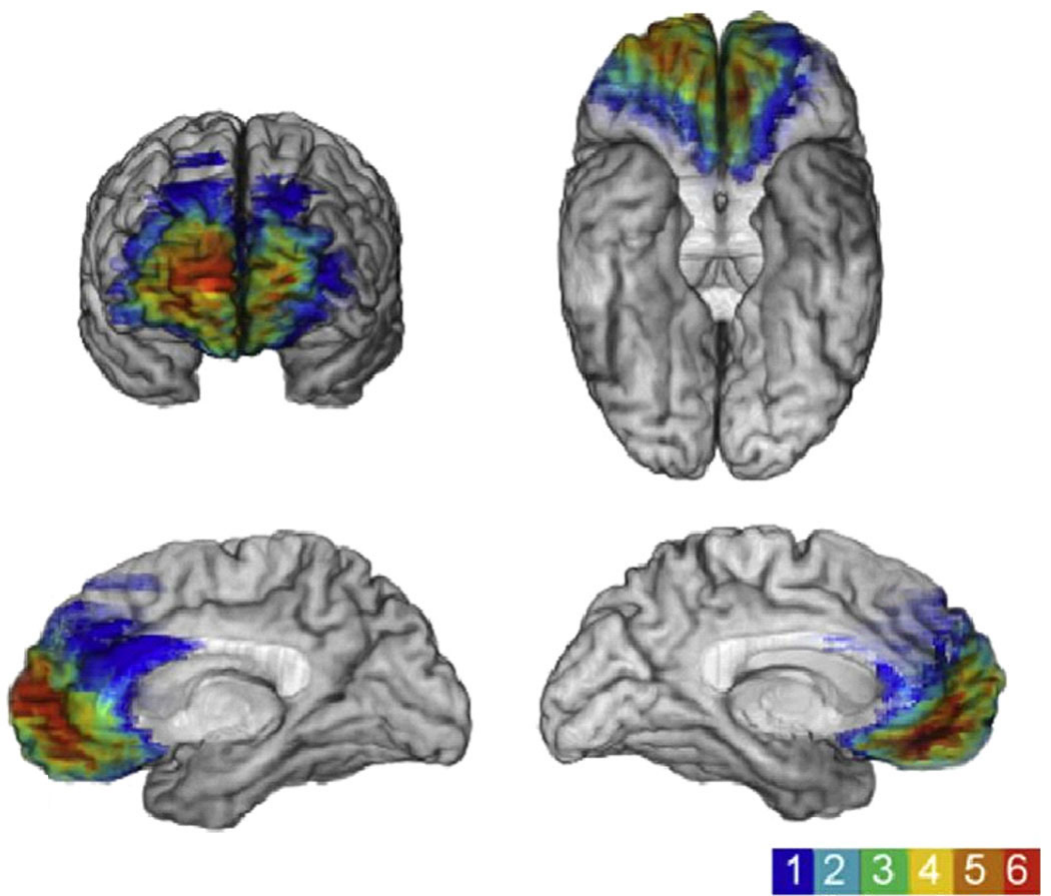
Data from six patients with medial frontal lesions are shown from Gupta *et al*. (2012; reproduced with permission from Elsevier). The colour bar to the lower right indicates the amount of overlap in the lesions, with red depicting the area of overlap across all patients. This maximal overlap region corresponds to the part of vmPFC that is usually engaged during fMRI in healthy participants when they recall autobiographical memories (see Fig. 7). Different views of the lesions are shown, as follows: upper left, a coronal view; upper right, a view of the underside of the brain; and lower left and right, medial sagittal views.

The fMRI and neuropsychological fields are replete with studies examining the vmPFC, associating it with a range of cognitive functions, including emotional processing (Goel & Dolan, 2003), planning (Burgess & Shallice, 1996), decision making (Shallice & Burgess, 1991; Bechara *et al*. 1998), moral behaviour (Thomas *et al*. 2011), schema (Tse *et al*. 2011; van Kesteren *et al*. 2013), valuation and reward processing (Jocham *et al*. 2012), risk taking and gambling (Bechara *et al*. 1994). While there have recently been some attempts to link the vmPFC and its role, for example, in decision making (Kumaran *et al*. 2009; Nieuwenhuis & Takashima, 2011) and reward (Barron *et al*. 2013) to memory and the hippocampus using fMRI, this has not involved autobiographical memory. More surprising still is the dearth of information about autobiographical memory in patients with bilateral vmPFC lesions (Fig. 8).

A complete review of the neuropsychological literature is beyond the scope of this article. To summarize with some examples from the literature, in the plentiful studies of patients with vmPFC lesions in decision making (e.g. Fellows, 2011) and gambling and reward studies (e.g. Bechara *et al*. 1994), memory scores are almost never reported. Where they are, it is often working memory scores or performance on standardized tests that assess memory within a 30 min time frame. There are reports that patients can describe the instructions or contingencies of tasks, suggesting intact memory (e.g. Rolls *et al*. 1994), but the status of their autobiographical memory and, in particular, their remote autobiographical memory, is never mentioned. In other studies, including those where autobiographical memory has been tested, vmPFC-damaged patients are typically included as part of a larger group of frontal-damaged patients, and so effects relating to vmPFC *per se* cannot be evaluated (e.g. Della Sala *et al*. 1993; Kopelman *et al*. 1999, 2003; Levine, 2004). In some cases, remote memory for public events has been tested, but not autobiographical memory (Mangels *et al*. 1996). Where autobiographical memory has been tested in the presence of bilateral vmPFC lesions, impairment has been noted (e.g. Levine *et al*. 1998; Piolino *et al*. 2003; Bird *et al*. 2004), but the lesions were not restricted to vmPFC and included shearing of white matter tracts or diffuse dementing pathologies.

Some clues about the vmPFC function might be gleaned from a related line of research concerning confabulation (Gilboa & Verfaellie, 2010). Patients who confabulate provide information that is verifiably false or that is clearly inappropriate for the context of retrieval, while being unaware of these falsehoods. Damage to vmPFC has been implicated in causing this disorder (Gilboa & Moscovitch, 2002). For instance, Moscovitch & Melo (1997) reported that patients with presumed damage or dysfunction in the region of the vmPFC confabulated in response to cues designed to elicit autobiographical memories. Interestingly, even when not confabulating, they had more difficulty than medial temporal lobe amnesic patients in recovering autobiographical memories related to these cues. There are numerous theories that seek to explain confabulation. For instance, some regard confabulation as temporal context confusion or an inability to filter information according to its relevance to ongoing reality (Schnider & Ptak, 1999; Nahum *et al*. 2009). Alternatively, strategic retrieval theories focus on investigating and characterizing how memory-monitoring processes break down in confabulation (Moscovitch 1989; Kopelman, 1999; Gilboa & Moscovitch, 2002), which may be related to another acknowledged difficulty that patients with frontal lobe lesions have in organizing information temporally (Moscovitch, 1989; Shimamura *et al*. 1990). These types of account seem to have relevance for understanding the role of the vmPFC in representing remote autobiographical memories. However, this must be tempered by the fact that confabulation is not associated only with vmPFC damage (Bajo *et al*. 2010), and many vmPFC-lesioned patients do not confabulate.

In summary, we lack fundamental knowledge about the status of autobiographical memory in patients with vmPFC lesions. Clearly, they are not densely amnesic in the manner of patients with bilateral hippocampal damage. Nevertheless, given vmPFC engagement during autobiographical memory retrieval in conventional fMRI studies, as well as our recent MVPA fMRI findings showing that information about remote autobiographical memories is represented there, there is an urgent need to know more. I suggest we should examine patients with more circumscribed frontal lobe lesions that either comprise only the vmPFC area described in this paper or specific subareas within it. Of course, patients with very specific frontal lesions restricted to locations of interest will be uncommon, but in the same way that rare patients with bilateral focal hippocampal damage are few but make important contributions to our understanding of memory, so it could be with small numbers of patients with focal damage to the ventromedial frontal lobes. They could illuminate the mechanisms of autobiographical memory and provide further insights into system-level memory consolidation. Moreover, they could elucidate the undoubted role of the vmPFC in other functions, such as value representations and reward processing. For instance, it could be that vivid remote memories are more valued and this is why the vmPFC is involved. In contrast, patients with vmPFC damage may have limited access to coherent representations of past experiences (or future scenarios, about which we also know very little in these patients), which could interact with their value-based decision making, rendering them unable to act in a well-informed and normative fashion.

## Concluding remarks

Autobiographical memories are dynamic and restless (Dudai, 2012) and, in particular, seem to undergo changes over time that involve the hippocampus and vmPFC. The MVPA of fMRI data is one of a number of multivariate approaches which now enable us to interrogate individual autobiographical memories in humans non-invasively and *in vivo* (Chadwick *et al*. 2012). The MVPA results have revealed some surprising findings that invite speculation, provoke numerous questions and suggest obvious targets for future investigations. The focus so far has been on vivid and easily retrievable memories. Studies examining autobiographical memories that vary in vividness, as well as the process of semanticization could provide a more complete picture of the system at work. In addition, exploring how memories develop, how long consolidation takes, the feature dimensions of autobiographical memories, how they are sustained, and how and why they sometimes decay (Hardt *et al*. 2013), in normal ageing and in the context of pathology (Bonnici *et al*. 2013*b*2013b), are important lines of inquiry that are now tractable. It will also be important to consider the computations that operate on memory traces and the nature of the connectivity between regions such as the hippocampus and vmPFC (Takashima *et al*. 2009; Soderlund *et al*. 2012). Overall, this decoding approach complements existing methods and may help to bridge the gap between functional neuroimaging and neuropsychology in humans, on the one hand, and electrophysiological and lesion studies in animals, as well as computational models of the hippocampus, on the other.

## Call for comments

Readers are invited to give their opinion on this article. To submit a comment, go to: http://ep.physoc.org/letters/submit/expphysiol;99/3/471.

## New Findings

- What is the topic of this review? This lecture is concerned with autobiographical memory representations, how they evolve and change over time, and the brain regions that support them.
- What advances does it highlight? The use of high resolution structural and functional magnetic resonance imaging combined with methods such as multi-voxel pattern analysis are opening up new opportunities to study memory consolidation in vivo in humans.
